# Martensite phase stress and the strengthening mechanism in TRIP steel by neutron diffraction

**DOI:** 10.1038/s41598-017-15252-5

**Published:** 2017-11-09

**Authors:** Stefanus Harjo, Noriyuki Tsuchida, Jun Abe, Wu Gong

**Affiliations:** 1 J-PARC Center, Japan Atomic Energy Agency, 2-4 Shirakata, Tokai-mura, Naka-gun, Ibaraki, 319-1195 Japan; 2 Graduate School of Engineering, University of Hyogo, 2167 Shosha, Himeji, Hyogo, 671-2280 Japan; 3 Present Address: Comprehensive Research Organization for Science and Society (CROSS), 162-1 Shirakata, Tokai-mura, Naka-gun, Ibaraki, 319-1106 Japan

## Abstract

Two TRIP-aided multiphase steels with different carbon contents (0.2 and 0.4 mass%) were analyzed *in situ* during tensile deformation by time-of-flight neutron diffraction to clarify the deformation induced martensitic transformation behavior and its role on the strengthening mechanism. The difference in the carbon content affected mainly the difference in the phase fractions before deformation, where the higher carbon content increased the phase fraction of retained austenite (γ). However, the changes in the relative fraction of martensitic transformation with respect to the applied strain were found to be similar in both steels since the carbon concentrations in γ were similar regardless of different carbon contents. The phase stress of martensite was found much larger than that of γ or bainitic ferrite since the martensite was generated at the beginning of plastic deformation. Stress contributions to the flow stress were evaluated by multiplying the phase stresses and their phase fractions. The stress contribution from martensite was observed increasing during plastic deformation while that from bainitic ferrite hardly changing and that from γ decreasing.

## Introduction

Transformation Induced Plasticity (TRIP) is well known as one of important effects in steel strengthening mechanism^1–6^. TRIP-aided multiphase steels (TRIP steels) contain several tens of percent of metastable retained austenite (γ) that transform to martensite (α′) during deformation, leading to the improvement of strength and ductility^1,2,4–6^. There have been many studies concerning the effects of phase fraction, stability of γ, and chemical composition to the TRIP behavior of TRIP steels^7–11^. The martensitic transformation behavior and the stability of γ have been reported to show strong relations with the strain rate and temperature^12–16^. The studies of the effect of carbon content have been also performed using TRIP steels containing carbon of 0.2 and 0.4 mass% by tensile tests, and have predicted that load partitioning to the γ was largely concerned in the difference in deformation induced martensitic transformation due to the different carbon content^15,16^. However, these studies remained unclear, because the qualitative prediction were interpreted only from the observation using the stress-strain curves as done in many studies^12–16^, and the quantitative measurements of phase stresses were not performed.

To understand the strengthening mechanism of multiphase steels during deformation, *in situ* neutron diffraction (ND)^17–19^ and X-ray diffraction (XRD) measurements^20,21^ during deformations have been confirmed as powerful tools. *In situ* ND studies during tensile deformation of TRIP and TWIP steels have been conducted using time-of-fight method^22–25^ and also angular dispersion method^26,27^. In previous reports^22,26^, the strengthening in TRIP steels was discussed mainly as a load partitioning only between bainitic ferrite matrix and γ, though the occurrence of martensitic transformation during deformation was confirmed from the decrease of phase fraction of γ. Because γ was found to be harder than bainitic ferrite, it was concluded that γ acts as an effective reinforcement responsible for large macroscopic stresses characteristic^22^. Meanwhile, Jacques *et al*.^27^, performed peak separations between bainitic ferrite and α′ by careful peak analyses on angular dispersion ND patterns with good statistics, and determined successfully phase stresses for bainitic ferrite, γ and α′. The α′ phase stress was found to be the largest, and its contribution to the strength was confirmed to be large enough though the phase fraction was small^27^. The phase fraction evolution was, however, not evaluated *in situ* during the deformation test for ND experiment. The phase fraction values measured using microscopy analysis and XRD in several deformed states were used to extrapolate the phase fractions to the deformation conditions of ND experiments. Lattice strains of α′ formed by the transformation were also successfully observed in a TRIP ultra-fine grained (TRIP-UFG) steel, when a neutron time-of-flight (TOF) diffractometer with high resolution was used for the measurement^24^. Decreases in the integrated intensities were observed in all γ-hkl peaks during Lüders deformation in the beginning of tensile deformation, where the deformation induced martensitic transformation preferentially occurred. Discussion concerning the validities of lattice strains and the quantitative evolution of phase stresses and phase fraction was however not performed.

In this study, tensile deformation behavior and deformation induced martensitic transformation in two TRIP steels with different carbon contents (Steel A and Steel B) and moderate grains (3~9 μm for ferrite and 1~4 μm for γ) (see Fig. S1 and Table S1) were studied by means of *in situ* TOF ND under continuous tensile deformation. The chemical compositions are shown in Table 1. The evolutions of textures, phase fractions, phase strains or phase stresses during deformation, and the contributions of constituent phases to the flow stress were evaluated to clarify the effects of carbon content on the deformation induced martensitic transformation and on the tensile properties. The validity of phase stresses observed by ND was also discussed as a composite model.

**Table 1:** Chemical compositions of TRIP steels (mass%).

Steel	C	Mn	Si	Al	P	S
Steel A	0.21	1.22	1.51	0.04	0.02	0.003
Steel B	0.41	1.18	1.47	0.04	0.02	0.003

## Results and Discussion

### Stress-strain curves

**Figure 1:**
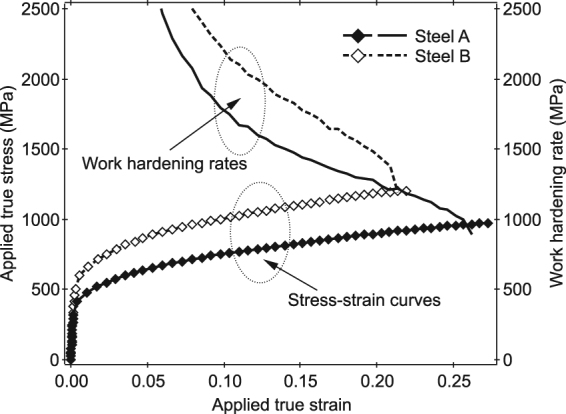
True stress–strain curves and work-hardening rates of Steel A and Steel B obtained from *in situ* neutron diffraction experiments during tensile deformation. The deformation in plastic region was conducted continuously without temporary stops. The true stress or true strain were evaluated from the nominal stress or nominal strain assuming uniform deformation within the gauge part of specimen.

Figure 1 shows the true stress-strain curves obtained from *in situ* ND experiments during tensile loading. The Lüders deformation that was reported in the TRIP-UFG steel^24^ was not observed, which might be due to the larger carbon concentration in γ (${X}_{{\rm{C}}}^{{\rm{\gamma }}}$) and the larger grain sizes in Steel A and Steel B. The increases in the applied strain monitored by two strain gauges glued separately in the parallel part of specimen were in linear relation to the increase in the crosshead displacement. These suggest that the deformation proceed almost uniformly in the parallel part of specimen of Steel A or Steel B. The true stress (*σ*_t_) or true strain (*e*_*t*_) shown in Fig. 1 were evaluated from the nominal stress (*σ*) or nominal strain (*e*) according to ${\sigma }_{t}=\sigma (1+e)$ or ${e}_{t}=\,\mathrm{ln}(1+e)$.

Because the tensile deformation in plastic regime was conducted continuously, the stress relaxation that is often observed in the *in situ* ND during tensile deformation with a displacement-stepwise manner, was suppressed. The larger carbon content led to increases in the elastic limit and the flow stress, while a decrease in the uniform strain. The work hardening rates inserted in Fig. 1 show the same shapes with different magnitudes, except for a drop in a large strain region (above 0.19) in Steel B. These show that, the martensitic transformation behavior in Steel A is similar to that in Steel B, though their carbon contents are different.

### Carbon concentration in austenite

Table 2 shows the phase fractions of γ (*f*_γ_) in Steel A and Steel B that were obtained from the Rietveld refinements of the ND patterns obtained before deformations. The *f*_γ_ values are in good agreement with those obtained from the OM and the XRD measurements. Distinctions between ferrite and bainite in the ND patterns were difficult, because they have the base-center-cubic (BCC) structure and the similar lattice constant. They were assumed to behave as one phase which is called bainitic ferrite (α) from here after. The refined lattice parameters before deformation can be used to estimate the ${X}_{{\rm{C}}}^{{\rm{\gamma }}}$ value according to the empirical equation^26,28^, as

1 $${X}_{{\rm{C}}}^{{\rm{\gamma }}}=({a}_{{\rm{\gamma }},0}\,-\,\frac{3.572}{2.8664}\,{a}_{{\rm{\alpha }},0})/0.033$$

**Table 2:** Phase fraction of γ (*f*_γ_) and carbon concentration in austenite (${X}_{{\rm{C}}}^{{\rm{\gamma }}}$) obtained from neutron diffraction.

	${{\boldsymbol{f}}}_{{\boldsymbol{\gamma }},{\bf{0}}}$ (%) by ND	${{\boldsymbol{X}}}_{{\bf{C}}}^{{\boldsymbol{\gamma }}}$ (mass%)	
		XRD	ND
Steel A	11.1	1.28^16^	1.32
Steel B	16.4	1.30^15^	1.36
TRIP-UFG^24^	19.5^24^	—	1.13^24^

ND: Neutron diffraction, XRD: X-ray diffraction.

Here, *a*_γ,0_ and *a*_α,0_ are the undeformed lattice constants of γ and α, respectively. The evaluated ${X}_{{\rm{C}}}^{{\rm{\gamma }}}$ values for Steel A and Steel B were close each other, as listed in Table 2, regardless of different carbon content. The higher carbon content might affect mainly to increases in the phase fractions of γ and bainite. In comparison to the TRIP-UFG steel^24^ having a carbon content similar to Steel B, the *f*_γ_ values in the specimens used in this study were much smaller, whereas the ${X}_{{\rm{C}}}^{{\rm{\gamma }}}$ values were larger.

### Evolutions of texture and phase fraction

**Figure 2:**
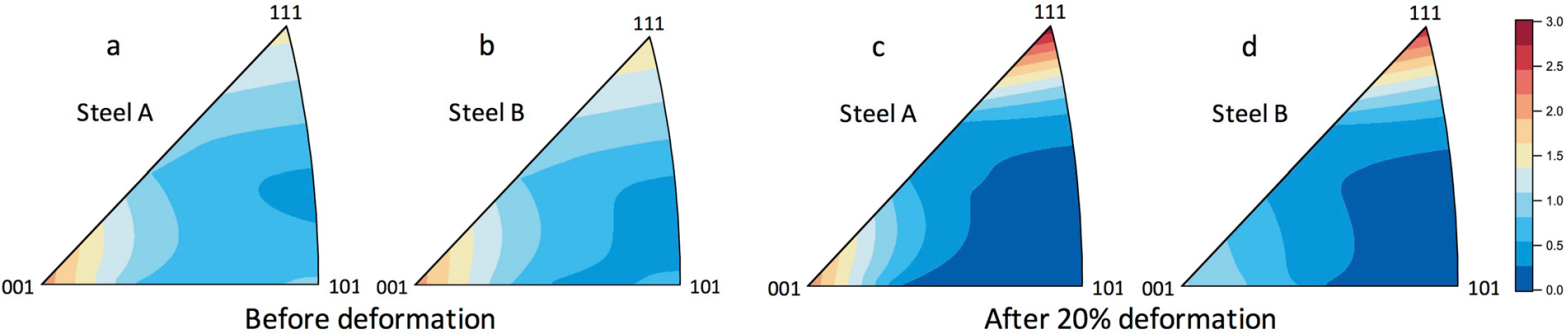
Inverse pole figure (IPF) images of γ in the axial direction (parallel to the rolling direction). (**a**) and (**b**) are the IPF images obtained before deformation. (**c**) and (**d**) are the IPF images after 20% deformation. (**a**) and (**c**) are for Steel A, and (**b**) and (**d**) are for Steel B.

Figure 2a and b show the inverse pole figures (IPF images) for γ measured before deformation. The IPF images were obtained from the ratios of integrated intensities of hkl peaks normalized to their crystal structure factors^29,30^. Weak rolling textures were already found before deformation in both specimens that might be introduced during the sample preparation. The IPF images in 20% deformed states (see Fig. 2c and d) show that the textures changed becoming the tensile deformation ones. The IPF images for α and α′ were not evaluated because the peak separation of α and α′ in many peaks at plastic deformations were difficult to conduct.

**Figure 3:**
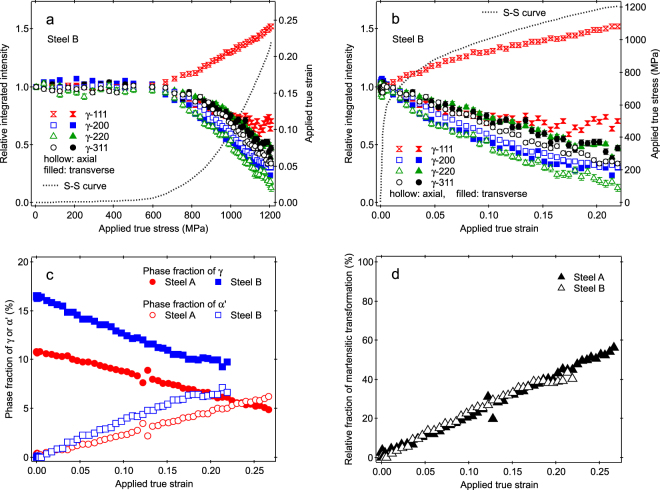
Relative integrated intensities, phase fractions, and relative fractions of martensitic transformation. (**a**) Relative integrated intensities of several γ-hkl peaks vs. applied stress in Steel B. (**b**) Relative integrated intensities of several γ-hkl peaks vs. applied strain in Steel B. (**c**) Phase fractions of γ and martensite vs. applied strain in Steel A and Steel B. (**d**) Relative fractions of martensitic transformation vs. applied strain in Steel A and Steel B.

Relative integrated intensities (*I*_rel_) were evaluated by normalizing the hkl integrated intensities obtained during deformation to those obtained before deformation and to specimen cross section reductions during deformation. Figure 3a and b show the *I*_rel_ values of several γ-hkl peaks for Steel B. The *I*_rel_ values were almost unchanged during deformation in elastic regime regardless of the <hkl> and specimen orientation (axial or transverse) (see Fig. 3a). They started to vary at the beginning of plastic deformation. The variations of *I*_rel_ values in plastic regime were different which depended on the <hkl> and specimen orientations. The *I*_rel_ value of γ-111 peak in the axial direction increased with increasing applied true stress or applied true strain, while the others decreased in different magnitudes. These show that the evolution of texture was accompanied during plastic deformation, being in good agreement with the IPF images in Fig. 2. The similar tendencies were also observed in Steel A (see Figure S2). The *I*_rel_ values of γ-311 peaks, which are known insensitive to the texture evolution in stable austenitic steels^30,31^, decreased in both the axial and transverse directions with the progress of plastic deformation. These are the evidences that the *f*_γ_ decreased as a result of martensitic transformation during plastic deformation. Meanwhile, in the TRIP-UFG steel, the *I*_rel_ value of γ-111 peak in the axial direction was reported to decrease also accompanied with the larger drops in other γ-hkl peaks during Lüders deformation indicating the occurrence of large amount of martensitic transformation^24^, but it gradually increases with the progress of deformation after the Lüders finished. The different magnitude of the decrease in γ peak intensity for different hkl suggests that the degree of martensitic transformation was different depending on the hkl due to the variant selection and/or the ununiform deformation among <hkl> orientations. The selection of hkl therefore might influence the accuracy of the evaluation of *f*_γ_ or the fraction of α′, because the hkl dependent martensitic transformation and the evolution of texture proceed in the same time. The variations of *I*_rel_ values of γ-311 might be used for the *f*_γ_ evaluation, but averaging of the *I*_rel_ values over all hkl peaks in the axial and transverse directions were used in this study.

Figure 3c shows the values of *f*_γ_ or phase fraction of α′ (*f*_α′_) evaluated in Steel A and Steel B. The *f*_γ_ values decreased almost linearly with respect to the applied true strain. The *f*_γ_ values remain much larger than zero though the specimens were broken. When the *f*_α′_ values were normalized to the *f*_γ_ value before deformation, relative fractions of martensitic transformation (*f*_mt,rel_) were evaluated and are plotted in Fig. 3d with respect to the applied true strain. The values of *f*_mt,rel_ for both specimens lay on the same line or curve, i.e., the increase rates of *f*_mt,rel_ to the applied true strain in Steel A and Steel B were the same, except values for Steel B in a large strain region above 0.19. The final *f*_mt,rel_ value was larger in Steel A (see Fig. 3d), though Steel B has larger carbon content. The smaller final value of *f*_mt,rel_ in Steel B might be due to the smaller grain size of γ or to the earlier fracture. The highest *f*_mt,rel_ value was however less than 60%. These results are in good agreement with other previous studies using microscopy analysis^27^, ND^26^ or XRD^16,32,33^, that not all γ transformed to α′ during tensile deformation.

### Evolution of lattice strain

The lattice strains (${\varepsilon }^{hkl}$) were estimated according to the equation as follows.

2 $${\varepsilon }^{{\rm{hkl}}}=\frac{{d}^{{\rm{hkl}}}\,-\,{d}_{0}^{{\rm{hkl}}}}{{d}_{0}^{{\rm{hkl}}}},$$

where ${d}^{hkl}$ is the lattice spacing obtained from the diffraction patterns during deformation and ${d}_{0}^{hkl}$ is the reference lattice spacing, respectively. The lattice strains to be discussed here after are those in the axial direction only, because the procedure of peak separation to determine the values of ${d}^{hkl}$ for α and α′ was difficult to perform on the patterns in the transverse direction. The lattice spacings of γ and α obtained before deformation were used as the ${d}_{0}^{hkl}$ values for γ and α, respectively. The α′ was not existed before deformation, and the ${d}_{0}^{hkl}$ values for α′ could be not easily determined from the diffraction data. Asoo *et al*.^24^ assumed that the ${d}_{0}^{hkl}$ value for α′ was similar to that for α. This approach might not be correct because carbon atoms were mainly concentrated in γ which transforms to α′ during plastic deformation, while α was almost carbon-free. Oliver *et al*.^25^ estimated the ${d}_{0}^{hkl}$ value for α′ from an α′ phase stress that was calculated using the stress balance among γ, α and α′ at an applied strain of the beginning of plastic deformation. This method might be applicable, but the obtained ${d}_{0}^{hkl}$ value depended on the applied strain state chosen for the stress balance calculation. Another way to estimate the average ${d}_{0}^{hkl}$ value for α′ is by measuring the residual phase stresses in unloaded states after plastic tensile deformation in many sample directions and suggesting the hydrostatic stress condition. However, in our case, the peak separations of α and α′ were possible only for diffraction patterns in the axial direction, and the residual phase stresses measurements in many sample directions couldn’t be applied. The simplest way might be the estimation of phase stresses of α′ by considering the stress balance among γ, α and α′ for the whole deformation without knowing the ${d}_{0}^{hkl}$ values for α′ and estimating the ${\varepsilon }^{hkl}$ values for α′. This kind of method was employed by Tomota *et al*.^18^ to evaluate phase stresses of cementite in a ferrite-cementite steel. However, to confirm the reliability of the peak separation procedure for determination of α and α′ peak positions, we need to estimate lattice strains in both α and α′. Here, we employed a relation of the lattice constant ratio between γ and α′ and their atomic densities^34^ to predict the ${d}_{0}^{hkl}$ values for α′, being similar to the method used by Saleh *et al*.^23^, as:

3 $${a}_{{\rm{\alpha }}^{\prime} }=\sqrt[3]{\frac{{\rho }_{{\rm{\gamma }}}}{2\,{\rho }_{{\rm{\alpha }}^{\prime} }}}\,{a}_{{\rm{\gamma }}},$$

where ${a}_{{\rm{\alpha }}^{\prime} }$ is the lattice constant for α′, and ${\rho }_{{\rm{\alpha }}^{\prime} }$ or *ρ*_γ_ is the atomic density in unit crystal for α′ or γ. The ${\rho }_{{\rm{\alpha }}^{\prime} }$ and *ρ*_γ_ can be calculated from the chemical composition according to empirical formulas^35^ as:

4 $${\rho }_{{\rm{\gamma }}}=8099.79\,-\,0.506\,T\,-\,(118.26\,-\,7.39\times {10}^{-3}\,T)\,{X}_{{\rm{C}}}^{{\rm{\gamma }}}\,-\,68.24\,{X}_{{\rm{Si}}}^{{\rm{\gamma }}}\,-\,6.01\,{X}_{{\rm{Mn}}}^{{\rm{\gamma }}},$$

5 $${\rho }_{{\rm{\alpha }}^{\prime} }=7875.96\,-\,0.297\,T\,-\,5.62\times {10}^{-5}\,{T}^{2}\,-\,(206.35\,-\,7.78\times {10}^{-3}\,T\,-\,1.472\times {10}^{-6}\,{T}^{2})\,{X}_{{\rm{C}}}^{{\rm{\alpha }}^{\prime} }\,-\,36.86\,{X}_{{\rm{Si}}}^{{\rm{\alpha }}^{\prime} }-7.24\,{X}_{{\rm{Mn}}}^{{\rm{\alpha }}^{\prime} },$$

where *T* is the temperature in °C, ${X}_{{\rm{C}}}^{{\rm{\gamma }}}$ or ${X}_{{\rm{C}}}^{{\rm{\alpha }}\text{'}}$ is the carbon concentration in γ or α′, ${X}_{{\rm{Si}}}^{{\rm{\gamma }}}$ or ${X}_{{\rm{Si}}}^{{\rm{\alpha }}\text{'}}$ is the silicon concentration in γ or α′, and ${X}_{{\rm{Mn}}}^{{\rm{\gamma }}}$ or ${X}_{{\rm{Mn}}}^{{\rm{\alpha }}\text{'}}$ is the manganese concentration in γ or α′. Since the α′ is formed by room temperature martensitic transformation from γ, $T=298\,{\rm{K}}\,(25{}^{\circ }{\rm{C}})$, ${X}_{{\rm{C}}}^{{\rm{\gamma }}}={X}_{{\rm{C}}}^{{\rm{\alpha }}\text{'}}$, ${X}_{{\rm{Si}}}^{{\rm{\gamma }}}={X}_{{\rm{Si}}}^{{\rm{\alpha }}\text{'}}$, and ${X}_{{\rm{Mn}}}^{{\rm{\gamma }}}={X}_{{\rm{Mn}}}^{{\rm{\alpha }}\text{'}}$ were assumed in the calculations. The ${X}_{{\rm{C}}}^{{\rm{\gamma }}}$ values by ND in Table 2 were adopted. The distribution of silicon or manganese in α and γ was tried to be observed in this study using an energy dispersive X-ray spectroscopy with the accelerating voltage of 20 kV, which was installed in a field emission scanning electron microscope (JEOL JSM-7100F). A visible difference in the distribution of silicon or manganese in α and γ was however not obtained (see Fig. S3). This result suggests that only the content of carbon was different in the α and γ, i.e., carbon might mostly concentrate in γ while less in α. The chemical compositions of silicon and manganese shown in Table 1 were therefore used as ${X}_{{\rm{Si}}}^{{\rm{\gamma }}}={X}_{{\rm{Si}}}^{{\rm{\alpha }}\text{'}}$ and ${X}_{{\rm{Mn}}}^{{\rm{\gamma }}}={X}_{{\rm{Mn}}}^{{\rm{\alpha }}\text{'}}$, respectively, in the equations (4) and (5). As results, the equation (3) can be rewritten to determine the lattice constant of undeformed α′ (${a}_{{\rm{\alpha }}^{\prime} ,0}$) from the ${a}_{{\rm{\gamma }},0}$ value, as:

6a $${a}_{\alpha \text{'},0}=0.8037(6)\,{a}_{\gamma ,0}\,{\rm{f}}{\rm{o}}{\rm{r}}\,\text{Steel A},$$

6b $${a}_{\alpha \text{'},0}=0.8039(6)\,{a}_{\gamma ,0}\,{\rm{f}}{\rm{o}}{\rm{r}}\,\text{Steel B}.$$

The values of ${d}_{0}^{hkl}$ for α′ were then calculated from the ${a}_{{\rm{\alpha }}\text{'},0}$ values estimated from the equation 6(a) or 6(b) for Steel A or Steel B, respectively. The reliabilities of the estimated ${d}_{0}^{hkl}$ values for α′ were also checked by comparing the residual phase stresses in unloaded states after plastically tensile deformation (see Fig. S4).

**Figure 4:**
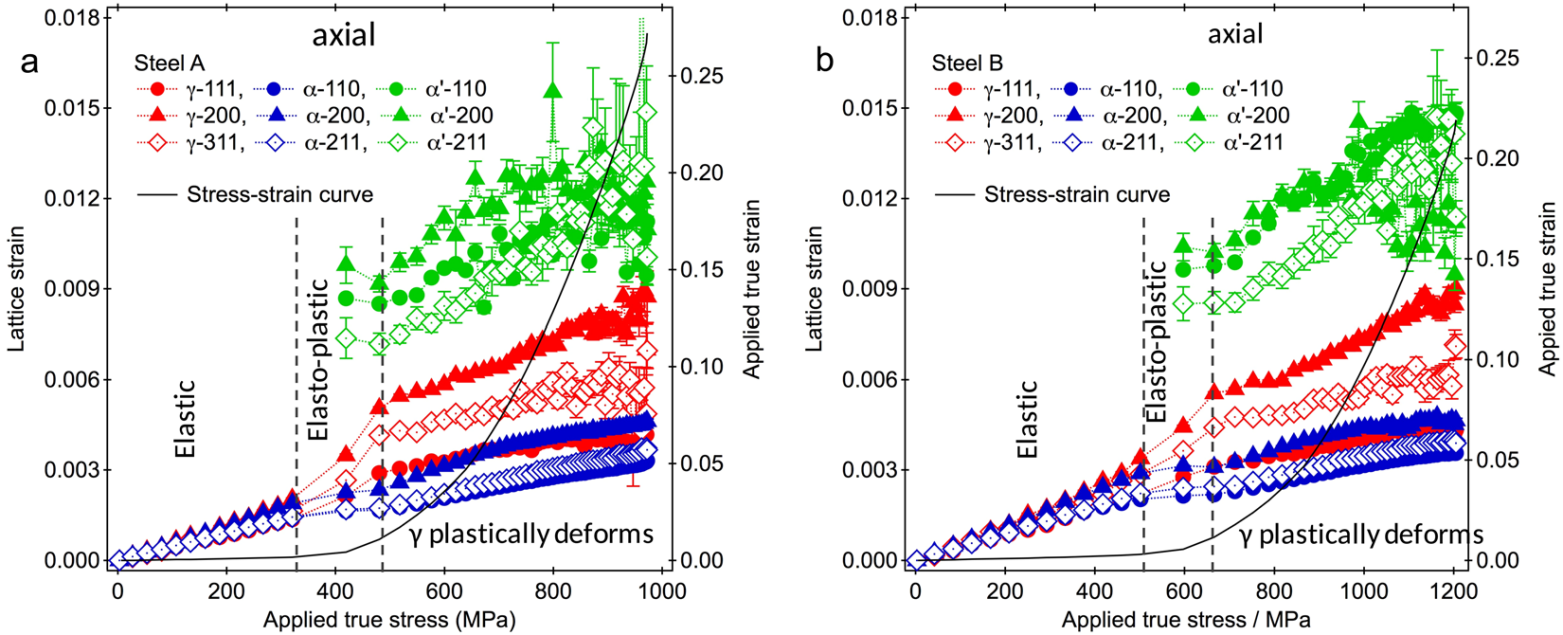
Lattice strains in the axial direction evaluated during deformation. (**a**) Steel A and (**b**) Steel B. The lattice strains for γ, bainitic ferrite (α) and martensite (α′) are collored with red, blue and green, respectively. The applied true stress–strain curves are superimposed. Dotted lines are plotted to indicate the changes of deformation stages.

Figure 4 shows results of ${\varepsilon }^{hkl}$ in the axial direction (${\varepsilon }_{11}^{hkl}$) obtained for three constituent phases in Steel A and Steel B. Three deformation stages were apparently determined from the ${\varepsilon }_{11}^{hkl}$ responses of γ or α to the applied true stress. The ${\varepsilon }_{11}^{hkl}$ responses of γ and α in elastic regime were linear, and the slopes were different depending on the constituent phases and <hkl> orientations. Steel B had a larger applied true stress limit of the linearity of ${\varepsilon }_{11}^{hkl}$ response than Steel A, which reflected the difference in the elastic limit in the stress-strain curve. The ${\varepsilon }_{11}^{hkl}$ responses of γ changed to have larger slopes in the early stage of plastic regime (elasto-plastic deformation stage), where α preferentially started to plastically deform. The ${\varepsilon }_{11}^{hkl}$ responses of α changed to have smaller slopes. These show that γ behaved as harder phase than α, which were in good agreement with those reported in refs^22,26,27^. The similar changes in the slope of lattice strain response were also confirmed in results of ${\varepsilon }^{hkl}$ in the transverse direction (see Fig. S5 for Steel A). The ${\varepsilon }_{11}^{hkl}$ values of α′ which were determined in the elasto-plastic deformation stage show much larger than those of other phases. These results show that the assumption of similar strains in α and α′ used for the peak fitting analyses of ND patterns in the previous works^22,26^ were erroneous. The slopes of ${\varepsilon }_{11}^{hkl}$ responses of γ and α changed again when γ also plastically deforms. The behavior of ${\varepsilon }_{11}^{hkl}$ of γ or α was similar to that studied using other TRIP steel^22,26,33^. Meanwhile, it was difficult to understand the behavior of ${\varepsilon }_{11}^{hkl}$ of α′ except of their large values, because of less number of data in the elasto-plastic deformation stage.

The intergranular strains were clearly observed in the ${\varepsilon }_{11}^{hkl}$ results of γ and α in Steel A and Steel B. During and after the elasto-plastic deformation stage, in α phase, the <100> grains families behaved as hard grains, the <110> grains families as soft grains, and the <211> grains families as moderate grains. In γ phase, the <100> grains families behaved as hard grains, the <111> grains families as soft grains, and the <311> grains families as moderate grains. These tendencies are in good agreement with the previous reports^22,24,26^. In α′ phase the intergranular strain behavior was, however, difficult to understand. The ${\varepsilon }_{11}^{hkl}$ values of α′ seem to slightly decrease or hardly change at the beginning after the α′ was formed (in the elasto-plastic deformation state) with increasing applied stress. These might be due to back stresses^36^ (kinds of phase stresses or intergranular stresses) generated by the transformation in the α′ grains.

### Phase stresses

Phase stresses can be evaluated from phase strains according to the Hooke’s law, using the elastic constants and the Poisson’s ratios. Due to the geometry of the tensile test, which involves ${\varepsilon }_{22}={\varepsilon }_{33}$ (with ${\varepsilon }_{11}$ along the tensile axis), two lattice strain components are sufficient to determine the mean stress level in each phase. When the transverse strain is not available, the mean stress level can be estimated as:

7a $${\sigma }_{{\rm{\gamma }},11}={E}_{{\rm{\gamma }}}\,{\varepsilon }_{{\rm{\gamma }},11}^{311},$$

7b $${\sigma }_{\alpha ,11}={E}_{{\rm{\alpha }}}\,{\varepsilon }_{\alpha ,11}^{211},$$

7c $${\sigma }_{\alpha ^{\prime} ,11}={E}_{{\rm{\alpha }}^{\prime} }\,{\varepsilon }_{\alpha ^{\prime} ,11}^{ave}.$$

Here, ${\sigma }_{{\rm{\gamma }},11}$, ${\sigma }_{{\rm{\alpha }},11}$ or ${\sigma }_{{\rm{\alpha }}^{\prime} ,11}$ is the phase stress in the axial direction for γ, α or α′, respectively. *E*_γ_, *E*_*α*_ or *E*_α′_ is the Young’s modulus for γ, α or α′, respectively. In this study, the values of ${\varepsilon }_{11}^{311}$ of γ (${\varepsilon }_{\gamma ,11}^{311}$) and ${\varepsilon }_{11}^{211}$ of α (${\varepsilon }_{\alpha ,11}^{211}$) were used to represent the phase strains for γ and α, respectively. The values of ${\varepsilon }_{11}^{311}$ and ${\varepsilon }_{11}^{211}$ are well known to display average macroscopic elastic strains for face-center-cubic^30,37,38^ and BCC^39,40^ polycrystalline materials, respectively. The values of ${\varepsilon }_{11}^{hkl}$ for α′ were averaged (${\varepsilon }_{{\alpha }^{\text{'}},11}^{ave}$) to determine the phase strain for α′. The values of ${E}_{{\rm{\gamma }}}=200\,{\rm{GPa}}$ and ${E}_{{\rm{\alpha }}}={E}_{{\rm{\alpha }}\text{'}}=210\,{\rm{GPa}}$ were used in the stresses calculations.

**Figure 5:**
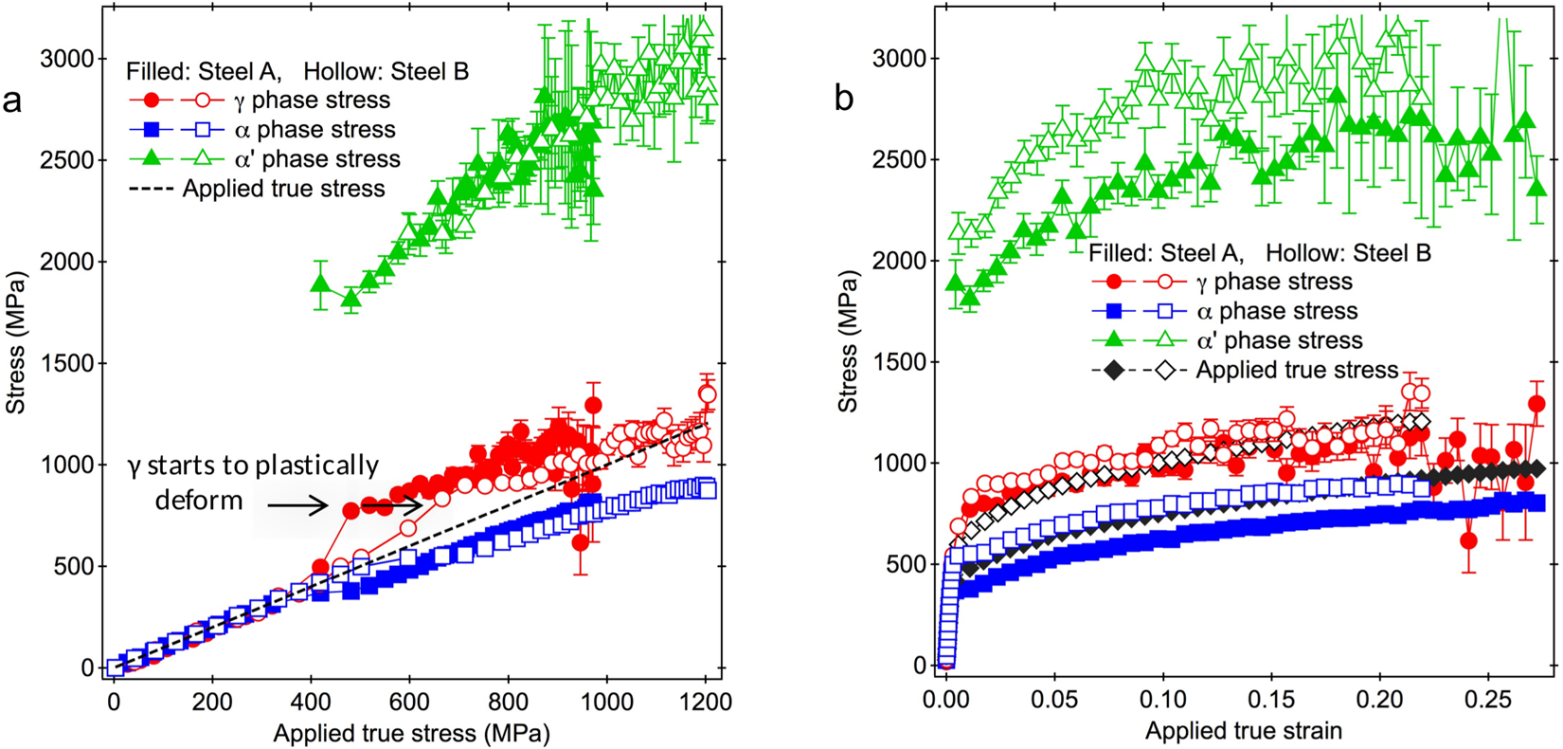
Phase stresses of γ, bainitic ferrite (α) and martensite (α′). (**a**) Phase stresses in Steel A and Steel B vs. applied stress. (**b**) Phase stresses in Steel A and Steel B vs. applied strain. The phase stresses of γ, α and α′ are collored with red, blue and green, respectively. The applied true stress–strain curves are superimposed.

Figure 5 shows the phase stresses, ${\sigma }_{\gamma ,11}$, ${\sigma }_{\alpha ,11}$ and ${\sigma }_{\alpha ^{\prime} ,11}$, in Steel A and Steel B. Steel A began entering the elasto-plastic deformation stage at a lower applied true stress than Steel B. The ${\sigma }_{\gamma ,11}$ value when γ started to deform plastically in Steel B was almost similar to that in Steel A (see Fig. 5a), regardless of the different elastic limits. The larger elastic limit in Steel B might be due to the larger *f*_γ_ that caused larger stresses to yield macroscopically. The ${\sigma }_{\gamma ,11}$ value when γ started to deform plastically can be understood also as a critical phase stress of γ needed to induce martensitic transformation (*CPSM*_γ_), because the appearance of α′ was confirmed at this stress level. The *CPSM*_γ_ values in both specimens were similar. These experimental results, with also considering that the increase rates of *f*_mt,rel_ to the applied true strain in Steel A and Steel B were similar (see Fig. 3d), opposed a qualitative prediction^16^ describing the smaller proportion of deformation induced martensitic transformation in a TRIP steel with lower carbon content as a result of the lower stress in γ, which was considered from the smaller phase fraction of γ.

The results in Fig. 5a or b show that the ${\sigma }_{\alpha ^{\prime} ,11}$ values were very large from the beginning (since the formation of α′ was identified). Steel B had larger ${\sigma }_{\alpha ^{\prime} ,11}$ values than Steel A in the whole range of applied true strain (see Fig. 5b). The ${\sigma }_{\alpha \text{'},11}$ values overlapped within error bars to be able for fitting in the same line or curve, when they were plotted as a function of the applied stress (see Fig. 5a). These show that the ${\sigma }_{\alpha \text{'},11}$ values in both specimens were in the same level for the same applied stresses. Steel B had a larger ${\sigma }_{\alpha \text{'},11}$ value at the beginning of plastic deformation (~2.1 GPa) than Steel A (~1.8 GPa) (see Fig. 5b), which could be considered because the deformation induced martensitic transformation in Steel B occurred at a higher applied stress level to exceed the *CPSM*_γ_ value. The stress levels of above 2 GPa for α′ are similar to those reported by Jacques *et al*.^27^. The high values of ${\sigma }_{\alpha \text{'},11}$ are accompanied with generation of a lot of dislocations in γ. The dislocation density in 10% tensile deformed Steel A evaluated from the ND pattern using the convolutional multiple whole profile procedure^41^ was about 2.7 × 10^15^ m^−2^. This dislocation density is much higher than that in a stable-austenitic AISI-316 steel at 12% tensile deformation^42^, 7.5 × 10^14^ m^−2^.

### Validation of phase stresses measured by ND

In many previous reports of *in situ* ND and XRD measurements during deformation of TRIP steels, the strengthening mechanisms were discussed mainly using the results of lattice strains, while the comparisons to the applied stresses have not been further performed^22,24,26,33^. The validities of the measured lattice strains were not confirmed, and the TRIP effect on the strength was not quantitatively discussed so far. In this study, to confirm the validities of lattice strains or phase stresses obtained above, contributed stresses from three phases (${\sigma }_{{\rm{\gamma }}}^{{\rm{cont}}}$, ${\sigma }_{{\rm{\alpha }}}^{{\rm{cont}}}$ and ${\sigma }_{{\rm{\alpha }}\text{'}}^{{\rm{cont}}}$) were calculated by multiplying the phase stresses and the phase fractions. The fraction-weighted average stress by diffraction (${\sigma }_{{\rm{Diff}}}$ or ${\sigma }_{{\rm{Diff}}}^{{\rm{w}}/{\rm{o}}\_{\rm{\alpha }}\text{'}}$), i.e., the stress balance, was subsequently calculated by summing the ${\sigma }_{{\rm{\gamma }}}^{{\rm{cont}}}$, ${\sigma }_{{\rm{\alpha }}}^{{\rm{cont}}}$ and ${\sigma }_{{\rm{\alpha }}\text{'}}^{{\rm{cont}}}$ as a composite model, using the next equations:

8 $${\sigma }_{{\rm{Diff}}}={\sigma }_{{\rm{\gamma }}}^{{\rm{cont}}}+{\sigma }_{{\rm{\alpha }}}^{{\rm{cont}}}+{\sigma }_{{\rm{\alpha }}\text{'}}^{{\rm{cont}}}={\sigma }_{{\rm{\gamma }},11}\,{f}_{\gamma }+{\sigma }_{{\rm{\alpha }},11}\,{f}_{\alpha }+{\sigma }_{{\rm{\alpha }}^{\prime} ,11}\,{f}_{{\rm{\alpha }}^{\prime} }\,{\rm{for}}\,{\rm{a}}\,{\rm{case}}\,{\rm{with}}\,{\rm{\alpha }}^{\prime} ,$$

9 $${\sigma }_{{\rm{Diff}}}^{{\rm{w}}/{\rm{o}}\_{\rm{\alpha }}\text{'}}={\sigma }_{{\rm{\gamma }}}^{{\rm{cont}}}+{\sigma }_{{\rm{\alpha }}}^{{\rm{cont}}}={\sigma }_{{\rm{\gamma }},11}\,(1-{f}_{{\rm{\alpha }}})+{\sigma }_{{\rm{\alpha }},11}\,{f}_{\alpha }\,\,{\rm{for}}\,{\rm{a}}\,{\rm{case}}\,{\rm{without}}\,\alpha ^{\prime} .$$

Here, *f*_α_ is the phase fraction of α which is assumed to be unchanged during tensile deformation, 88.9% for Steel A and 83.6% for Steel B, and ${f}_{\alpha }=1-{f}_{\gamma }-{f}_{\alpha \text{'}}$.

**Figure 6:**
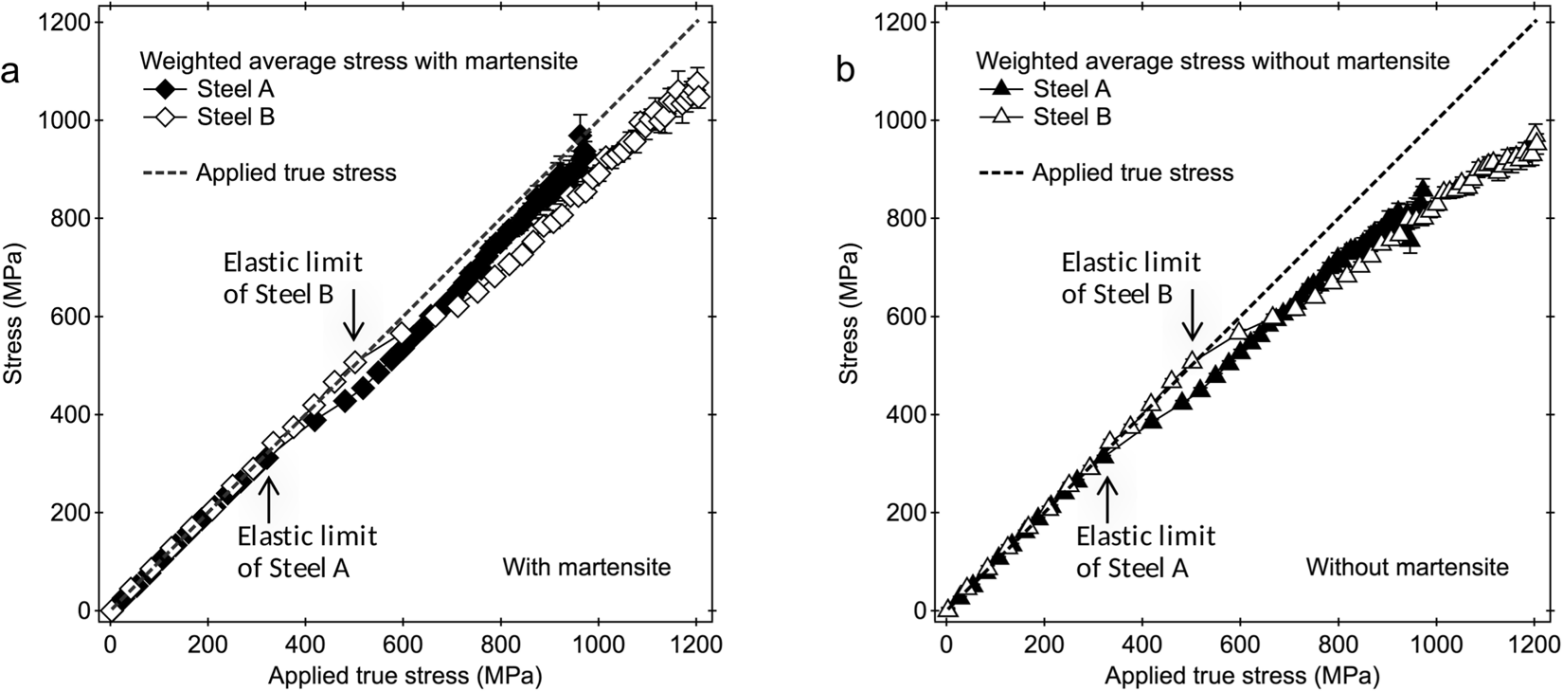
Fraction-weighted average stresses by diffraction vs. the applied true stress. (**a**) The fraction-weighted average stresses by diffraction were calculated with considering the contribution of martensite. (**b**) The fraction-weighted average stresses by diffraction were calculated without considering the contribution of martensite.

The results are summarized in Fig. 6a and b. In elastic regime, the ${\sigma }_{{\rm{Diff}}.}$ or ${\sigma }_{{\rm{Diff}}}^{{\rm{w}}/{\rm{o}}\_{\rm{\alpha }}\text{'}}$ values for Steel A and Steel B were in good agreement with the applied stresses. The ${\sigma }_{{\rm{Diff}}}$ (see Fig. 6a) deviated from the applied stress to have a slightly lower value at the beginning of plastic deformation, but it became close again or was kept to have the same gradient to the applied stress. The largest stress differences were only about 11% for Steel A and 13% for Steel B. The ${\sigma }_{{\rm{Diff}}}^{{\rm{w}}/{\rm{o}}\_{\rm{\alpha }}\text{'}}$ (see Fig. 6b), however, started to deviate from the applied stress at the beginning of plastic deformation and continuously to have larger deviation with the deformation. These results show that the stress contribution from α′ should be taken into account in the strengthening mechanisms of these specimens, and opposed the previous assumption that the strengthening in the TRIP steels was mainly described only by the load partitioning between α and γ^22,26^.

The lattice strains and phase stresses measured and evaluated in this study were however those only for the axial direction due to the limitation of ND and the data analyses. To analyze more correctly including the back stresses and the shear stresses, strain measurements and analyses for various directions are preferable, which need more advanced methods.

### Contribution of α′ to the flow stress

**Figure 7:**
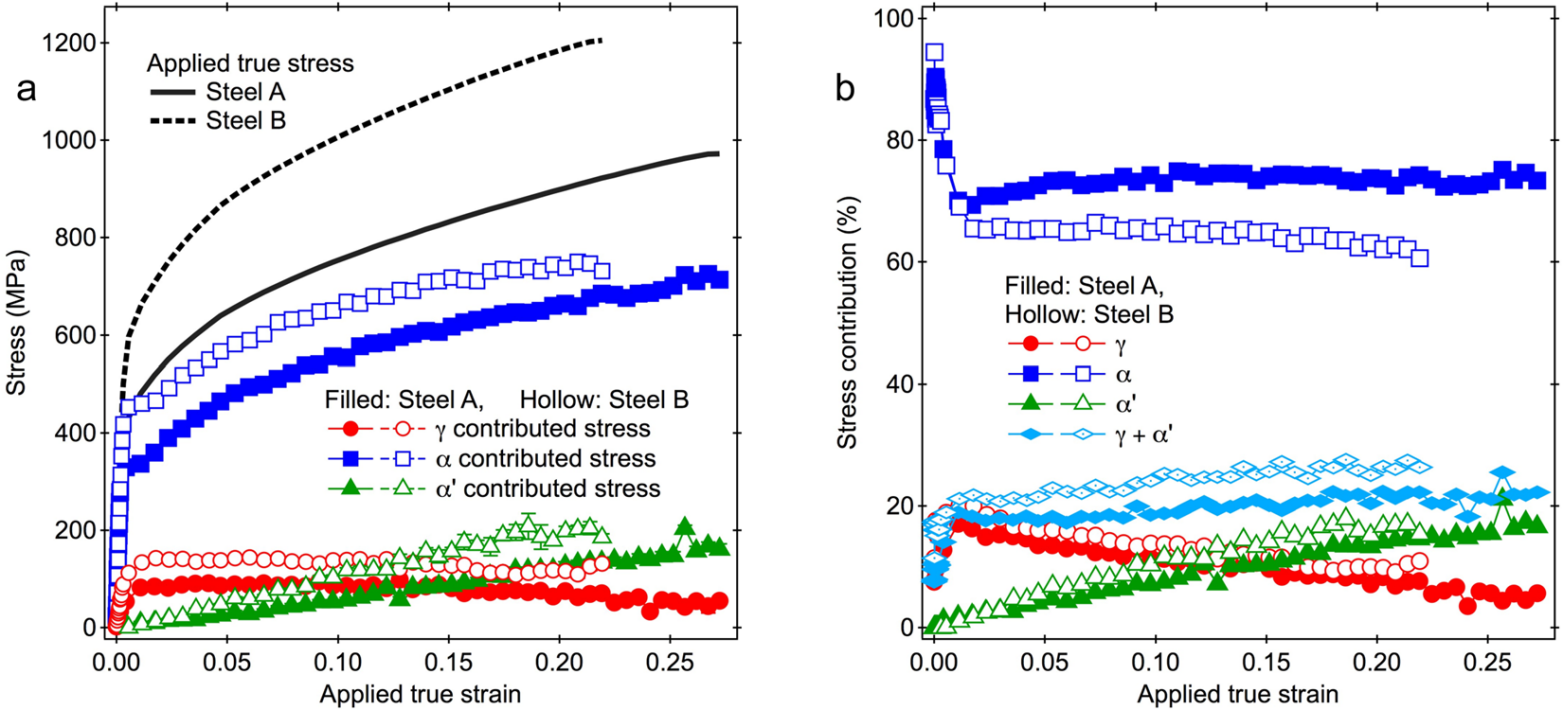
Contributed stresses and stress contributions. (**a**) Contributed stresses from three phases in Steel A and Steel B during deformation. (**b**) Stress contributions to the flow stress from three phases in Steel A and Steel. Contributed stresses and stress contributions were obtained by normalization of the contributed stresses to the applied stresses. Those for γ, bainitic ferrite (α) and martensite (α′) are collored with red, blue and green, respectively.

The values of ${\sigma }_{{\rm{\gamma }}}^{{\rm{cont}}}$, ${\sigma }_{{\rm{\alpha }}}^{{\rm{cont}}}$ and ${\sigma }_{{\rm{\alpha }}\text{'}}^{{\rm{cont}}}$ in Steel A and Steel B are plotted and compared with the applied true stresses in Fig. 7a. Steel B had larger ${\sigma }_{{\rm{\alpha }}}^{{\rm{cont}}}$ values than Steel A. The difference in ${\sigma }_{{\rm{\gamma }}}^{{\rm{cont}}}$ values between Steel B and Steel A became much larger than the difference in ${\sigma }_{\gamma ,11}$ shown in Fig. 5b. Steel B had a larger increase rate of ${\sigma }_{{\rm{\alpha }}\text{'}}^{{\rm{cont}}.}$ to the applied true strain than Steel A, because Steel B had the larger values of ${\sigma }_{{\rm{\alpha }}\text{'},11}$ and *f*α′ for the same applied true strains than Steel A. These can be understood because Steel B had the larger *f*_γ_ value before deformation than Steel A. The increase rate of ${\sigma }_{\alpha \text{'}}^{{\rm{cont}}}$ to the applied strain in Steel B became slightly smaller in the large strain region above 0.19, due to the smaller increase rates of *f*_mt,rel_ shown in Fig. 3d. The ${\sigma }_{\alpha \text{'}}^{{\rm{cont}}}$ increase in the strain region above 0.19 is considered to relate with the drop of work hardening rates in Steel B in the same strain region (see Fig. 1).

When the values of ${\sigma }_{{\rm{\gamma }}}^{{\rm{cont}}}$, ${\sigma }_{{\rm{\alpha }}}^{{\rm{cont}}}$ and ${\sigma }_{{\rm{\alpha }}\text{'}}^{{\rm{cont}}}$ were normalized to the applied stresses, stress contributions to the flow stress (in percent) could be evaluated as shown in Fig. 7b. In the elastic regime, there were only the stress contributions from α and γ to the flow stress, and their total contributions in Steel A and Steel B were almost similar. At the beginning of plastic deformation (applied true strain below 0.02), the stress contribution from α became lower, while that from γ became oppositely higher in both specimens, showing that α preferentially yielded than γ. The sum of stress contributions from γ and α in this deformation stage was not 100%, because α′ was generated and probably degraded some accuracy of peak fitting of α and α′. In the higher applied true strain region, the stress contribution from α in Steel A increased slightly and then was kept almost constantly with increasing applied true strain, whereas those in Steel B unpredictably decrease gradually. The stress contributions from α′ were low at the beginning of plastic deformation. They increased with increasing applied true strain due to the increase in *f*_α′_. When the applied true strains reached about 0.15 and 0.14 for Steel A and Steel B, respectively, the stress contributions from α′ became higher than those from γ. These applied strain values of 0.14–0.15 correspond to the *f*_mt,rel_ values of about 30% for both steels (see Fig. 3d). When the stress contributions from γ and α′ were summed, they were found to increase gradually with increasing applied true strain.

The results and discussion explained above demonstrated directly that the deformation induced martensitic transformation in TRIP steels contribute to the increase in strength. However, the lattice strains and the integrated intensities obtained in this study are difficult to understand the plastic strain in each constituent phase, and the discussion of TRIP effect on the ductility needs assistance using different methods.

## Conclusions

Deformation induced martensitic transformation behavior in TRIP steels with different carbon contents (0.2 and 0.4 mass%) were studied using *in situ* time-of-flight ND experiments during tensile tests. Main results obtained are as follows.

1. The different carbon content affected mainly the phase fractions of bainite and γ, while the carbon concentration in γ was kept to be similar.
2. The martensitic transformation started at the beginning of plastic deformation, and continuously occurred during plastic deformation accompanying with texture evolutions in the constituent phases.
3. The changes in the relative fraction of martensitic transformation with respect to the applied strain in both steels were found to be in linear relation with the same gradient regardless of different carbon content.
4. The phase stress of α′ was found much larger than that of γ or α since the α′ was formed. The stress contribution from α′ to the strength increased with increasing applied true strain, where that from α was almost unchanged and that from γ decreased, demonstrating directly that the deformation induced martensitic transformation contribute to the increase in strength.

## Methods

### Specimen preparation, microscopy analysis and X-ray diffraction

Two TRIP steels, consisting of different carbon content, were used in this study. The steels were prepared by vacuum melting, heat-treatment at 1473 K for 1.8 ks, hot rolling to 8 mm thickness, and cold rolling to 2.2 mm thickness. The steels were then solution treated at 1073 K for 180 s, air cooled to 998 K, rapid cooled to 673 K and subsequently held for 180 s, and then air cooled to RT, to produce microstructures containing ferrite, bainite and γ. Specimens used in this study were made from the steels after removal of 0.2 mm thick parts from both surfaces.

### Neutron diffraction

Plate specimens with the parallel part of 55 mm long, 6 mm wide and 1.8 mm thick were prepared for *in situ* ND experiments during tensile deformation at room temperature. The tensile loading direction was adjusted being parallel to the rolling direction. To monitor the applied strain and the localized deformation that might occur, two strain gauges with 2 mm gauge length were glued on both sides of 6 mm wide surfaces in such a way that they were separated each other for about 25 mm. The experiments were conducted at TAKUMI^43,44^, a high resolution and high intensity TOF neutron diffractometer for engineering materials science at Materials and Life Science Experimental Facility of Japan Proton Accelerator Research Complex. The specimen was mounted horizontally in a loading machine which was installed at TAKUMI, in such a way that the ND patterns in the axial and transverse directions were measured simultaneously using two detector banks with the scattering angles of ±90° (see Fig. S6a). The ND data in each detector bank was integrated over ±15° horizontal-width and ±15° vertical-width. The gauge size was determined using an incident slit size of W5 × H5 mm^2^ and radial collimators viewing 5 mm wide, i.e., an average data from the center area of parallel part was obtained. The tensile deformation was conducted in a step-load controlling manner with 300 s holding in elastic regime, and in a continuous manner with a constant crosshead speed with an initial strain rate of 1.8 × 10^−5^ s^−1^ in the plastic regime. Diffraction patterns for the plastic regime were then extracted periodically with 300 s accumulating time for different applied strain or stress values.

### Neutron diffraction data analyses

Data analyses were performed by a single peak fitting method and a multi peak fitting method using a Rietveld software called Z-Rietveld^45^. Phase strains and macroscopic lattice strains during loading tests are often estimated using average lattice constants determined from the Pawley method or Rietveld refinement^23,37^, but sometimes the intergranular stresses are difficult to be absorbed^38^ and the positions of peaks with large intensities become dominant contributions to the lattice constants^30^. In this study, the evaluation of lattice strain was performed by a single peak fitting using the Z-Rietveld, and three peaks were selected for each constituent phase. A convolution function of the pseudo-Voigt function and the rise-decay function was used to fit the peak profile (see Fig. S6b). Peak separation between α and α′ was conducted by assuming that the c/a ratio of α′ was constant during whole deformation and could be simplified by a single-broad BCC peak. The similar procedures have been conducted in previous XRD^46^ and ND studies^19,24^. The parameters of rise-decay function were fixed to keep the instrumental peak shape to be similar for α and α′, while the parameters of pseudo-Voigt function were refined during fitting. These achievements were not only because of the high resolution of neutron diffractometer used in this study, but also because of the high beam intensity that realized continuous plastic deformation to suppress the stress relaxation in the plastic regime. However, the peak separations were possible only for the patterns in the axial direction. The peaks of α and α′ in the patterns in the transverse direction became close each other due to the Poisson’s effect, and the peak separations became difficult to perform.

## Electronic supplementary material

Supplementary information
