# Supplementary material for: Martensite phase stress and the strengthening mechanism in TRIP steel by neutron diffraction
Source: Sci Rep. 2017 Nov 9;7:15149. doi: 10.1038/s41598-017-15252-5 (PMC5680272; doi:10.1038/s41598-017-15252-5)
Supplement: Supplementary file 1 — Supplementary information [file 41598_2017_15252_MOESM1_ESM.pdf]

Supplementary Figures & Table:

Martensite phase stress and the strengthening mechanism in TRIP steel by neutron diffraction

Stefanus Harjo, Noriyuki Tsuchida, Jun Abe, Wu Gong

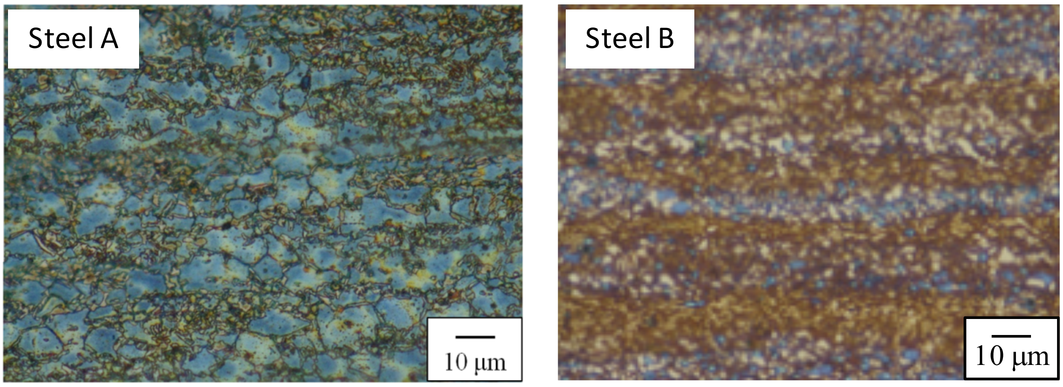

Figure S1

Optical microscopy images of Steel A and Steel B. The OM observations were performed after a tint etching using a mixture of the three ingredients 4% nital, 7% picral, and saturated aqueous sodium thiosulfate. In the image for Steel A, ferrite, bainite and retained austenite (γ) are collored with blue, brown and light-yellow, respectively. In the image for Steel B, ferrite, bainite and γ are collored with blue, brown and white, respectively. The horizontal direction is parallel to the rolling direction.

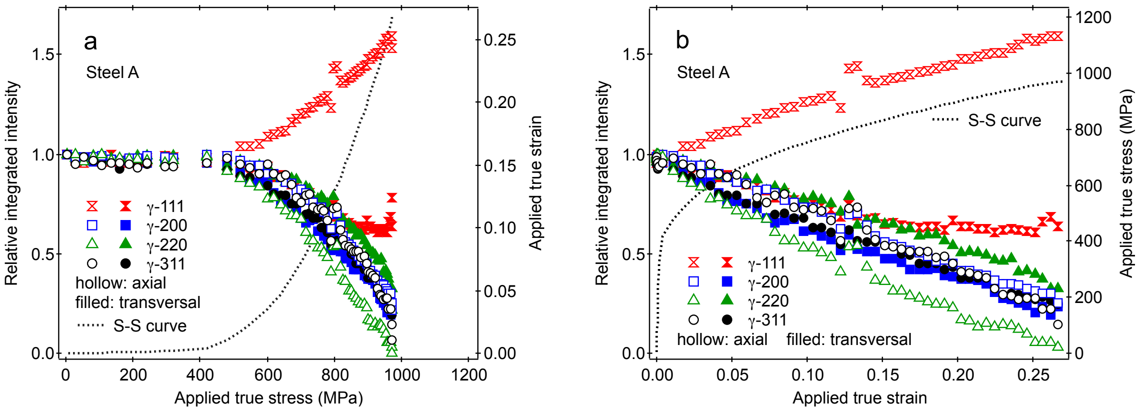

Figure S2: Relative integrated intensities (I_{rel}) in Steel A

(a) The I_{rel} values of several γ-hkl peaks vs. applied stress. (b) The I_{rel} values of several

γ-hkl peaks vs. applied strain. Being similarly to Fig. 3a and 3b for Steel B, the I_{rel} values were almost unchanged during deformation in elastic regime regardless of the <hkl> and specimen orientation (axial or transverse). They started to vary at the beginning of plastic deformation. The variations of I_{rel} values in plastic regime were different which depended on the <hkl> and specimen orientations. The I_{rel} value of γ-111 peak in the axial direction increased with increasing applied true stress or applied true strain, while the others decreased in different magnitudes. These show that the evolution of texture was accompanied during plastic deformation.

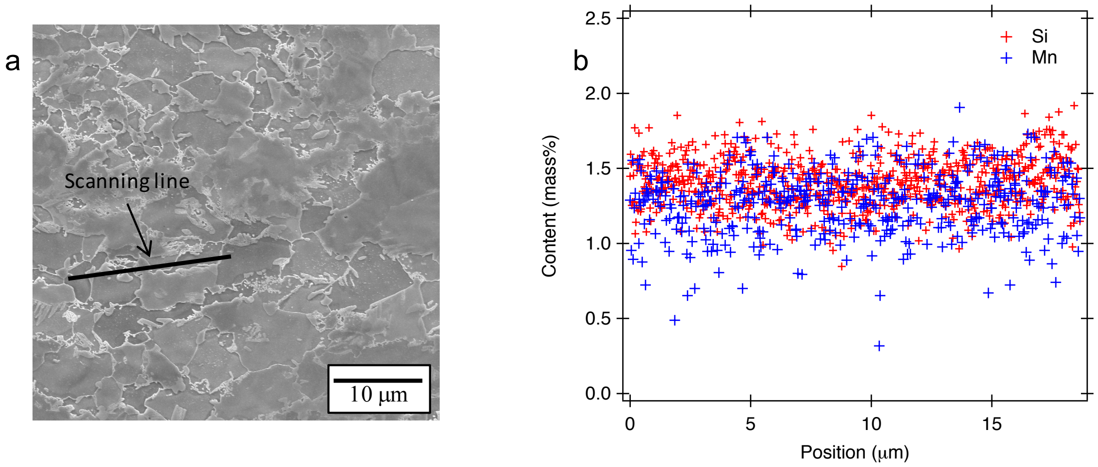

Figure S3

(a) Scanning electron microscopy image of Steel B and a scanning line showing the positions used for the measurement of distribution of silicon or manganese using energy dispersive X-ray spectroscopy.

(b) Distribution of silicon or manganese (in mass%) measured by the energy dispersive X-ray spectroscopy with the accelerating voltage of 20 kV, with respect to the position in the scanning line in A. The content of silicon or manganese hardly changed with changing the position which indicated the different phase (ferrite, bainite or γ). This result suggests that the distributions of silicon or manganese in ferrite, bainite and γ were similar.

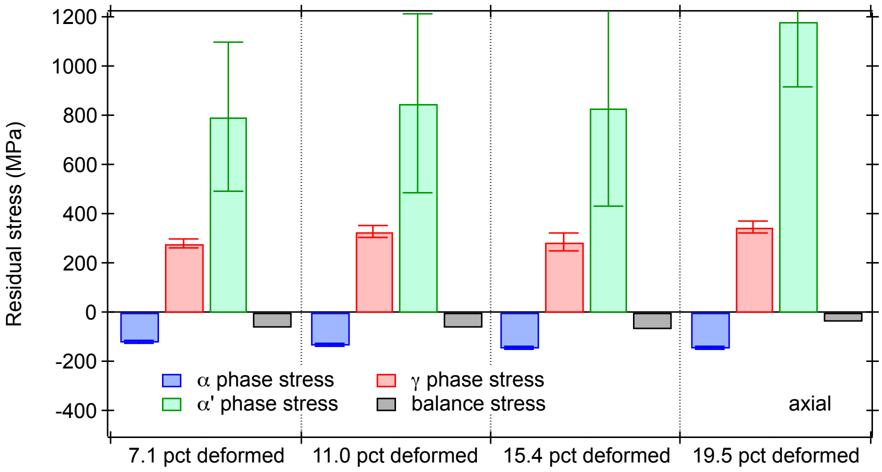

Figure S4

Residual phase stresses and balances of residual phase stresses in Steel A in unloaded states after plastically tensile deformation in axial direction.

The balance stress was evaluated according to the composite model. The residual stress in α was compressive and the residual stresses in γ and α' were tensile. The residual stresses in α' were very high, but the balances of residual stresses in the unloaded states of all conditions were close to zero and they were slight with negative values. If the residual stress in α' is over-estimated, the balance stress must be a positive value. The values of balance stresses in four different conditions were also similar, indicating the high reliabilities of the phase stresses and the d0 values.

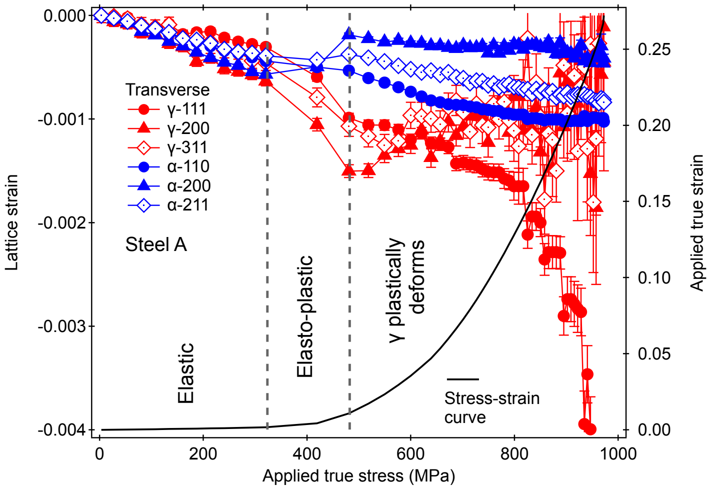

Figure S5

Lattice strains in the transverse direction evaluated during deformation of Steel A. The lattice strains for γ and bainitic ferrite (α) are collored with red and blue, respectively. The applied true stress–strain curves are superimposed. Dotted lines are plotted to indicate the changes of deformation stages.

The lattice strains for α' could not be evaluated, because the peak separations of α and α' were difficult for diffraction patterns in the transverse direction. The α peaks had much larger intensities than the α' ones and the lattice strains were consequently considered to be mainly for α, because the volume fraction of α was much larger than the α'. Three deformation stages were apparently determined from the transverse lattice strain responses of γ or α to the applied true stress. The transverse lattice strain responses of γ and α in elastic regime were linear, and the slopes were different depending on the constituent phases and <hkl> orientations. The transverse lattice strain responses of γ changed to have larger slopes in the early stage of plastic regime (elasto-plastic deformation stage), where α preferentially started to plastically deform. The transverse lattice strain responses of α changed to have smaller slopes. These show that γ behaved as harder phase than α, being consistent with the results in the axial direction in Fig. 4. When γ deformed plastically, the γ peaks in the transverse direction become much broader than those in the axial direction due to the large intergranular strains in the transverse direction. This created ambiguity in peak position of γ, and the lattice strains for γ at applied stresses larger than 600 MPa might not be accurate.

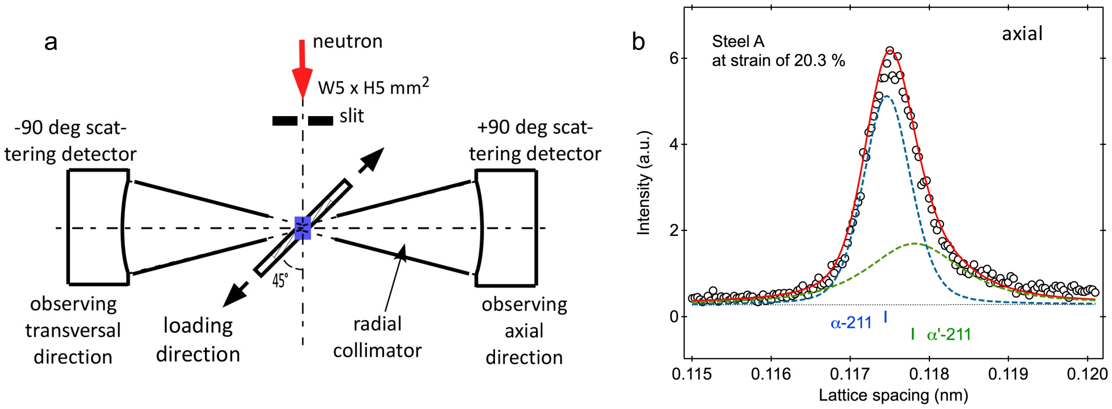

Figure S6

(a) Schematic drawing of in situ neutron diffraction experimental setup. The specimen was mounted horizontally in a loading machine which was installed at TAKUMI, in

such a way that the ND patterns in the axial and transverse directions were measured simultaneously using two detector banks that have the scattering angles of ± 90°. The instrumental resolution was tuned to 0.3 %.

(b) A typical fitting appearance using double peak contribution on diffraction peaks of α-211 and α'-211 in the axial direction of Steel A taken at deformation of 0.20. A successful separation was achieved.

Table S1

Phase fractions and grain sizes of parent phases before deformation in Steel A and Steel B. The phase fractions were estimated using an optical microscopy (OM) analysis or X-ray diffraction (XRD) with Cu-Kα, and the grain sizes of ferrite and γ were estimated using the OM analysis.

Steel	Phase fraction (%)			Grain size (μm)	
	Ferrite (OM)	Bainite (OM)	γ (OM/XRD)	Ferrite	γ
Steel A	67.6	21.8	10.6	8.9	~ 1
Steel-B	28.2	55.8	16.0	3.1	4.2

OM: Optical microscopy, XRD: X-Ray diffraction
